# Consumer Behaviour to Be Considered in Advertising: A Systematic Analysis and Future Agenda

**DOI:** 10.3390/bs12120472

**Published:** 2022-11-23

**Authors:** Ahmed H. Alsharif, Nor Zafir Md Salleh, Shaymah Ahmed Al-Zahrani, Ahmad Khraiwish

**Affiliations:** 1 Azman Hashim International Business School, Universiti Teknologi Malaysia, Skudai 81310, Johor, Malaysia; 2 Department of Economic & Finance, College of Business Administration, Taif University, Taif 21944, Saudi Arabia; 3 Department of Marketing, Faculty of Business, Applied Science Private University (ASU), Amman 11931, Jordan

**Keywords:** PRISMA, neuromarketing, advertising, WoS database, brain processes

## Abstract

In the past decade, neurophysiological and physiological tools have been used to explore consumer behaviour toward advertising. The studies into brain processes (e.g., emotions, motivation, reward, attention, perception, and memory) toward advertising are scant, and remain unclear in the academic literature. To fill the gap in the literature, this study followed the Preferred Reporting Items for Systematic Reviews and Meta-Analyses (PRISMA) protocol to extract relevant articles. It extracted and analysed 76 empirical articles from the Web of Science (WoS) database from 2009–2020. The findings revealed that the inferior frontal gyrus was associated with pleasure, while the middle temporal gyrus correlated with displeasure of advertising. Meanwhile, the right superior-temporal is related to high arousal and the right middle-frontal-gyrus is linked to low arousal toward advertisement campaigns. The right prefrontal-cortex (PFC) is correlated with withdrawal behaviour, and the left PFC is linked to approach behaviour. For the reward system, the ventral striatum has a main role in the reward system. It has also been found that perception is connected to the orbitofrontal cortex (OFC) and ventromedial (Vm) PFC. The study’s findings provide a profound overview of the importance of brain processes such as emotional processes, reward, motivation, cognitive processes, and perception in advertising campaigns such as commercial, social initiative, and public health.

## 1. Introduction

Self-report has been adopted in marketing activities to evaluate and identify consumer responses toward stimuli in the marketing sector, such as advertising practices. According to Carrington, et al. [1], the self-report methods reflect/measure the conscious responses (e.g., perception, approach/withdrawal attitudes, and thoughts) toward advertising. In fact, self-report cannot measure the unconscious or subconscious responses, which represent the majority of consumer responses, such as decision-making. Thus, researchers and marketers have adopted neuroscience tools such as electroencephalography (EEG) in the marketing field, to better understand the unconscious responses of consumers [2,3,4], which has led to an emerging new field, the so-called “Neuromarketing”. Smidts [5] was the first business researcher who coined the term “neuromarketing”, in 2002. Neuromarketing is a hybrid field containing numerous areas/fields such as psychology, marketing, and neuroscience [6]. According to Fortunato, et al. [7], the thanks for spreading this term was given to the Bright House Company when it created the first fMRI laboratory for marketing research.

In the hyper-competitive environment, neuromarketing is a mainstay in advertising because it has an opportunity to gauge consumers’ neural responses as emotional responses toward advertising; thereby, it is a revolutionary field for a better understanding of the subconscious and unconscious consumer responses. According to the literature, the rapid technological progress in neuroscience technology led to a better understanding of consumers’ behaviour in several contexts, such as, but not limited to, advertising [8,9,10,11]. Therefore, marketing and advertising leaders have used this technology to boost innovation and success in marketing and advertising, by controlling and minimizing task conflict [12]. According to Ramsoy [13], neuromarketing tools have been divided into four clusters, as follows: (1) neuroimaging tools such as electroencephalography (EEG), magnetoencephalography (MEG), functional magnetic resonance imaging (fMRI), positron emission tomography (PET), and transcranial magnetic stimulation (TMS); (2) physiological tools such as galvanic skin responses (GSR), eye-tracking (ET), electrocardiogram (ECG), and electromyography (EMG); (3) self-report methods such as surveys, observations, focus groups, and interviews; (4) behavioural measurements, such as the implicit association test (IAT). For instance, neuroimaging tools have been used to gauge emotions, attention, and memory regarding advertising [14,15]. At the same time, physiological tools have been used to gauge the physiological responses of consumers, such as, but not limited to, visual fixation in-store at the purchasing point, thereby providing valuable and fruitful insights into the attitudes of consumers (i.e., approach, withdrawal) [16,17,18]. Behavioural measurements are used to measure the reaction time of consumers toward stimuli, and self-reporting is used to measure the conscious behaviour of consumers toward stimuli such as approach/withdrawal attitudes [18].

According to the literature, the first fMRI investigation in neuromarketing was carried out by McClure, et al. [19], and largely contributed to the practical studies of neuromarketing [18]. Therefore, neuromarketing research has received attention from both academia and the industrial world as a means of filling the gap in traditional marketing methods and overcoming the limitations by reducing consumer social-bias (e.g., consumer choices can be affected by others) [7]. However, understanding the global trends in advertising research within the neuromarketing field is still unclear in academic studies. In addition, to date, no investigation has determined the current neurophysiological and physiological techniques that have been used in studying the unconscious/subconscious responses of consumers toward advertising such as YouTube video scenes, TV ads, public health ads (antismoking), initiative ads (encouraging the use of seat belts in cars). To sum that, this study tries to analyse the extracted articles in depth, to provide a precise and concise conclusion. The contributions of this work are summarized as follows:

- Provides a profound evaluation of the current advertising research that has been used to investigate unconscious and subconscious consumer behaviour, such as emotional dimensions, perceptions, reward processes, and approach/withdrawal motivation toward advertising.
- Provides an overview of the current neurophysiological and physiological tools that were used in advertising within the neuromarketing context between 2009 and 2020.

In this vein, the current paper provides an inclusive overview of neuromarketing research and the current research objectives. Section two presents the methodology and data-collection process. Section three presents the growth of the publication, topics of interest and a thematic analysis. A discussion of the study’s findings is presented in Section four. Section five presents a concise conclusion and the implications of our work. Finally, Section six presents the limitations and future directions.

## 2. Materials and Methods

This review study is designed to select empirical articles from the Web of Science (WoS) database in advertising within the neuromarketing context, to fill the gap in the literature. The reason behind choosing the WoS database over Scopus is the availability of cleaner data, which means reducing the duplications as compared to the Scopus database; additionally, WoS includes publications in top-tier journals [20]. The first step was to follow the instructions of the PRISMA protocol to select the empirical articles which used neuroimaging and physiological tools to investigate consumer responses to advertising research within neuromarketing [21]. The reason behind the use of the PRISMA protocol to select the relevant articles for this study were that it has been widely used in social science and business to extract and select articles related to the study, for example, online learning [22], neuromarketing [23], and service and healthcare [24,25].

For the second step, we used the content analysis of selected empirical articles for this study. The above processes will provide us with a profound insight into the advancement in advertising research by identifying and analysing the general and specific areas. Additionally, providing us with a better understanding of advertising research that used neuroimaging and physiological tools/methods and which can be considered when we are conducting further research into advertising research. Therefore, the findings of this study provide a guide for new scholars who are interested in advertising research within a neuromarketing field.

**Figure 1:**
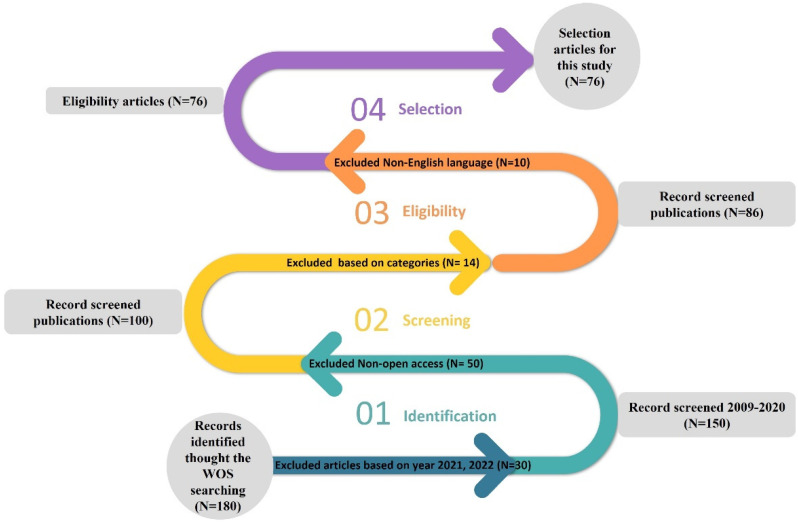
PRISMA protocol to extract empirical articles for this systematic study.

Relevant empirical articles have been selected by using the following query applied to the title, abstract, and keywords: (((“neuromarketing” OR “consumer neuroscience”) AND (“advertising” OR “advertisement”) AND (neuroimaging OR physiological))). This study extracted 76 empirical articles relevant to this review paper from January 2009 to December 2020. This study focused on empirical journal-articles, in comparison to conferences and book chapters, which generally undergo a much more rigorous review process and therefore improve the credibility of published research in journals [26]. Figure 1 demonstrates the selection of articles processed for this study, as follows:

- Methods: neuroimaging and physiological tools;
- Publication year: January 2009 to December 2020;
- Language: English;
- Document type: original articles (chapters of books, articles from conferences, reviews, and proceedings books were excluded).

## 3. Results

### 3.1. Growth of the Publication

**Figure 2:**
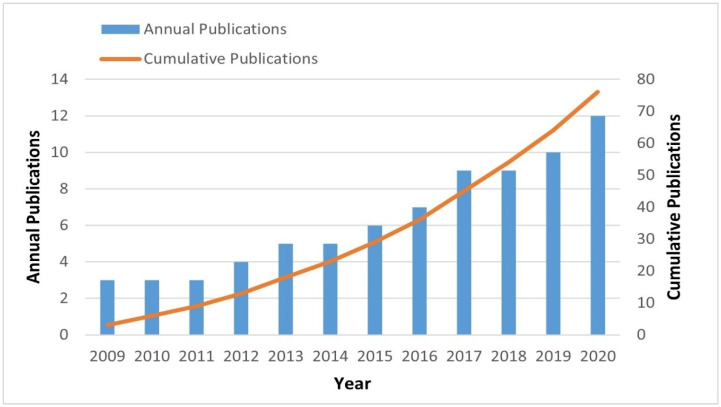
PRISMA protocol to extract empirical articles for this systematic study.

Seventy-six articles were extracted from the WOS which used neuromarketing tools. According to the literature, McClure, et al. [19] published the first neuromarketing study in 2004, when they investigated the neural correlates of consumer behaviour (e.g., choice, decision-making) toward two brands (Coca-Cola vs. Pepsi Cola). However, the first investigation into advertising was carried out by Morris, et al. [27]. They found the gyri regions of the brain were highly related to pleasure/displeasure and high/low arousal. From January 2009 to December 2020, there was a fluctuation in the number of published articles in advertising. In 2020, it was the highest number of annual publications with thirteen articles, as depicted in Figure 2.

By exploring the relevant articles to develop this review article, it was possible to classify the global academic-research trends and advancement in advertising and neuromarketing, as follows: (i) neuroimaging and physiological tools used in advertising; and (ii) consumers’ brain processes to be considered in advertising, such as emotions, motivation, reward process, attentions, perception, and memory. By reviewing the selected articles, we can enrich our understanding, to achieve the objectives of this study. Table 1 shows the summary of the neuroimaging and physiological tools that were used to investigate the brain processes of consumers toward advertising research.

### 3.2. Topics of Interest and Thematic Analysis

#### 3.2.1. Neuroimaging and Physiological Tools Used in Advertising

We found that the neural-response recording-tool most used in advertising was EEG. EEG is an electrical and also a non-invasive technique to gauge the unconscious/subconscious responses of consumers toward ads by recording the voltage changes of frequencies at scalp regions [28,29,30,31]. According to [32], there are five frequency bands (e.g., delta, theta, alpha, beta, and gamma). The EEG tool uses a 10–20 global system, which is used to describe the electrode locations on the volunteer’s scalp, for example, (Fp), (F), (P), (O), (C), and (T) describe the prefrontal, frontal, parietal, occipital, central, and temporal, respectively. Moreover, it uses the same number of electrodes on both side of the volunteer’s head (i.e., right and left side) [33,34]. In addition, it has an excellent temporal accuracy (estimated in milliseconds (ms)) and a poor spatial accuracy (estimated at 1 cm^3^ at the scalp regions) [35,36,37]. In addition, it is not expensive or noisy [38]. fMRI and fNIRS are non-invasive, metabolic tools. Both are used to record oxygenated and deoxygenated haemoglobin in the brain [39,40]. fMRI has a superior spatial accuracy (estimated at 1–10 mm^3^ of the deep structure of the brain) compared with fNIRS, which has poor spatial accuracy (estimated at 4 cm of cortical-activity regions) [41]. Meanwhile, both have acceptable temporal accuracy (estimated in seconds) [42,43]. fMRI and fNIRS have been used in marketing research to record the subconscious/unconscious responses of consumers (e.g., preference, perceptions, purchase decisions, choices) toward marketing stimuli [41,43,44]. fNIRS is a portable, novel, promising, and silent neuroimaging technique, which is also cheaper than fMRI [39,45,46].

ET is a portable technique that is used to gauge physiological reactions such as eye movements, pupil dilation, saccade, and fixation toward the stimuli of marketing [18]. According to Hoffman [47], it is used for measuring eye movements and the attention of consumers, which is beneficial for psychology and neurological research. Eye fixations last between 200 and 350 ms while reading a text and watching video scenes, respectively, while 200 ms refers to the duration of saccadic eye-movements [48]. The set of fixations and saccades is named the scan route, and analyses visual perception and cognitive purpose [49]. Pupil dilation with a longer blink-period gives us better information about processing [18]. The GSR and ECG tools are used to gauge the emotional reactions of consumers toward ads [50]. For example, sweating level is recorded by the GSR tool and the heart rate/heartbeat is measured by the ECG tool [50,51,52]. In addition, both of them can measure the autonomic nervous system and evaluate the internal emotional status of consumers [53]. Therefore, GSR and ECG are convenient and reliable techniques for measuring the emotional status of consumers and changes in skin conductance, respectively [54]. IAT is able to identify the customers’ attitudes toward marketing stimuli such as brands or ads (e.g., like/dislike) by recording the reaction time of customers [18]. EMG is used to measure the reactions shown on individuals’ faces (e.g., pleasure/displeasure, …, etc.) toward advertising [55], because facial-expression analysis is significant for marketers and advertisers, because faces can provide beneficial information about perceptions of customers toward ads in terms of measuring visible and invisible facial-muscle reactions [29].

#### 3.2.2. Brain Processes to Be Considered in Advertising

##### Emotion and Feelings

The feeling is a relatively conscious aspect of emotional status [56], which derives from individuals’ judgments such as level of pleasure or unpleasure toward advertising [31]; it is likely the best way to understand and explain the physiological responses of the consumer toward ads [31,57]. Many research studies have affirmed that ad-elicited feelings are strong indexes of consumers’ response toward advertising [58]. On the opposite side of the spectrum, emotion is an unconscious aspect of emotional status which correlates to an automatic somatic response such as increased heart beat in some conditions (fright, anger) [56,59], which is crucial for making decisions, learning, and solving problems [60]. Advertisers and marketers can use both in advertising to captivate consumers’ attention, thereby enhancing purchase intention. Emotions are accompanied with changes in the autonomic nervous system (i.e., zygomatic facial muscles, corrugator facial muscles, and heart-rate), which can provide rich information about the emotional status of consumers. Therefore, the study of emotions has attracted many researchers and advertisers [61].

**Figure 3:**
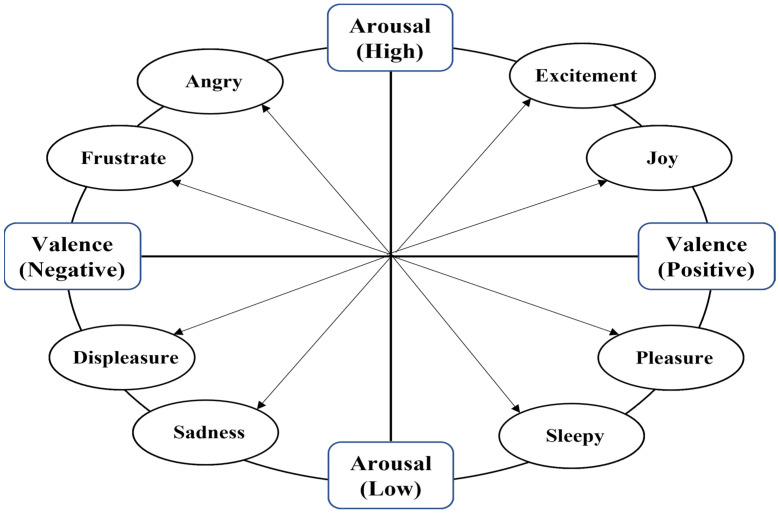
Dimensions model of emotions [68].

Emotion is constructed from a neural network in the brain, which performs basic psychological activities/functions (e.g., memory, perception, salience detection) [62]. Therefore, emotion is defined as the set of changes in the individual’s physiological and subconscious and unconscious responses, based on the individual’s experiences [63,64]. In addition, emotion is a relationship between humans and the environment, including multiple subcomponents (e.g., physiological, behavioural, appraisal, and expression [65]. The cognitive and neurological frameworks of the role of emotion in decision-making has been investigated more through Damasio’s theory, which is also known as the somatic marker hypothesis [66,67]. Consequently, researchers have agreed on two dimensions for measuring emotional responses toward stimuli: (i) valence/balance (i.e., pleasure or displeasure, depression or excitement), (ii) arousal (e.g., high or low) (Figure 3) [28,68,69]. Figure 3 shows that the valence indicates either positive or negative emotional-status which is evoked by external stimuli such as advertising. In addition, valence is measured from the positive to the negative side. On the other side of the spectrum, arousal indicates the level of excitement; whether high or low, it is measured from high to low levels [68,70,71].

Table 2 shows several methods to measure emotions and feelings toward marketing stimuli. Previous studies have used, for example, self-report and physiological methods to analyse the emotional responses of consumers toward advertising [72]. For example, the EMG and self-report investigation of Lajante, et al. [73] gauge the consumer’s pleasure/displeasure toward advertisements. The findings of the experiment revealed that pleasure/displeasure had positively impacted the attitudes of customers toward advertisements. The ECG, EDA, and questionnaire study of Baraybar-Fernández, et al. [50] found that audio and visual ads with sad messages have more influence on participants. Barquero-Pérez, et al. [53] used the ECG, EDA and questionnaire in their experiment. They found that each type of ad generated a different emotion, such as surprise, anger, and so forth. In addition, physiological tools have been used to investigate the effectiveness of online advertising (e.g., YouTube) [51]. For example, Guixeres, et al. [51] used brain response, ECG, and ET to investigate the relationship between ad effectiveness and the number of views on YouTube. They found that there is a solid relationship between ad effectiveness and the number of views on YouTube. Herrador, et al. [74] conducted an EDA experiment and the findings revealed that both groups of participants (male and female) indicated a strong initial activation; moreover, they noticed a reduced activation during the most significant part of video material in the male group. Venkatraman, et al. [28] used several neuromarketing tools to evaluate participant’ responses to a 30-second TV advertisement. The findings revealed that the activity in the ventral striatum could be the predictor of response to advertising. Grigaliunaite and Pileliene [75] conducted an experiment by using ET and they found that emotional and rational advertising appeals influence consumers’ responses in various ways. The IAT and ET investigation of Pileliene and Grigaliunaite [76] found that warm-colour temperature attracts more visual attention to the advertisement; thereby generating a positive implicit attitude and inducing the buying intentions toward the advertised product, compared with cool-colour temperature advertisements, whether the spokesperson is a female or male celebrity. Similarly, Grigaliunaite and Pileliene [77] found that negative smoking images reflected a negative implicit attitude/behaviour of individuals toward those images and smoking behaviour, increasing the influence on individuals’ intention to whether to quit or not to start smoking. The ERP, ET, and questionnaire investigation of Pileliene and Grigaliunaite [78] found that a well-known female spokesperson has a significant impact on the effectiveness of fast-moving consumer-goods advertising.

There has been an interesting growth in understanding the non-verbal responses of emotional status toward advertising by using neuroscience methods such as fMRI, EEG, fNIRS [31,51,79,80,81,82]. For example, Plichta, et al. [45] conducted an fNIRS experiment to investigate the detection of sensory activity by measuring emotional signals in the auditory field. The findings revealed that pleasurable/unpleasurable sounds increased the activity in the auditory cortex, compared to neutral sounds. Gier, et al. [41] conducted the fNIRS experiment to explore whether the fNIRS tool had the ability to predict the success of point-of-sale elements by measuring the neural signals of brain regions such as the dlPFC. The findings revealed that the fNIRS has the ability to predict the success elements at the point of sale, relying on the cortical-activity effect.

The EEG investigations of Vecchiato, et al. [83], and Vecchiato, et al. [84] found that activity in the right frontal alpha is associated with pleasure/liking ads, while the left frontal alpha correlated to displeasure/disliking ads. Additionally, Vecchiato, et al. [85] found that there were gender (i.e., male and female) differences in interest toward commercial categories and scenes in two ads. The EEG experiment of Harris, et al. [86] found that emotional advertisements are more effective than rational advertisements, which leads to a positive change in decision-making, inducing donation, and liking. The findings of Chen, et al. [87] revealed that e-cigarette ads increased the smoking desire; additionally, e-cigarette ads increased activity in the left middle-frontal-gyrus, the right medial-frontal-gyrus, the right parahippocampus, the left insula, the left lingual gyrus/fusiform gyrus, the right inferior-parietal-lobule, the left posterior-cingulate, and the left angular-gyrus. Wang, et al. [88] and Royo González, et al. [89] found that the narrative approach of ads and exposure to branding products have a favourable influence on the consumers’ preferences and excitement. The fMRI investigations of Morris, et al. [27] and Shen and Morris [90] found that pleasure and displeasure are correlated with more activity in the inferior frontal- and middle temporal-gyrus, respectively, while low and high arousal is associated with the right superior-temporal- and right middle-frontal-gyrus, consecutively. Leanza [91] used EEG and found that some of the emotive features of the virtual reality (VR) experience significantly influenced consumers’ preferences. Ramsoy, et al. [92] found that arousal and cognitive load were highly connected to subsequently stated travel-preferences; moreover, consumers’ subconscious emotional and cognitive responses are not identical to subjective travel-preference. Shestyuk, et al. [93] found that the EEG is a convenient tool to predict the success of TV programs and determine cognitive processes. Wang, et al. [94] conducted an experiment to propose a generative-design method using EEG measurements. The findings revealed that the product image that was generated with preference EEG-signals had more preference than the product image generated without preference EEG-signals. Kim, et al. [95] conducted an experiment to identify the effect of visual art (e.g., Mondrian’s and Kandinsky’s artworks) on consumers’ preferences, by using EEG. The findings showed that the visual effects induced high emotional arousal, which might promote heuristic decision-making. Mengual-Recuerda, et al. [96] found that food served by a chef positively influences emotions, while dishes with special presentations attract more attention than traditional dishes. The EEG study of Eijlers, et al. [31] found that arousal is positively connected to prominent ads in the wider population and negatively to consumer attitudes toward these ads.

##### Motivation

According to Lang and Bradley [97], emotions and motivation processes are highly intersected and correlated. Chiew and Braver [98] and Pessoa [99] found that cognition and consumers’ behaviours are highly affected by motivational processes. For example, positive motivational stimuli will urge individuals toward achieving goals (e.g., obtain or predict a reward by performing a task correctly) [100], while negative motivational stimuli can lead to distraction [101].

Pessoa [102] and Raymond [103] suggested that motivational processes are a compass of consumers’ attitudes toward external stimuli to engage with the environment and achieve goals. Higgins [104] suggested two dimensions for measuring motivational processes such as withdrawal and approach attitudes. Researchers and practitioners attempted to investigate the neural responses of motivational processes to better understand consumers’ behaviours toward advertisements and products [83]. For example, Cherubino, et al. [105] carried out an experiment using EEG to investigate the relationship between the PFC and motivational dimensions. The findings revealed that the PFC is related to motivational dimensions, wherein the right PFC correlated to withdrawal attitudes and the left PFC related to approach attitudes. The EEG investigations of Pozharliev, et al. [106] and Zhang, et al. [107] recorded the brain responses of subjects toward luxury products (motivations). The findings showed that social motivations have a vital role in influencing the purchase of luxury products in order to satisfy social goals (at least one goal). The EEG investigation of Bosshard, et al. [108] found that liked brands reflect more motivational aspects and activity signals in the right parietal-cortices than disliked brands.

Therefore, there is a strong relationship between the activation of the PFC and motivational dimensions toward marketing stimuli such as advertisements [109]. Therefore, marketing researchers and practitioners have to focus on the motivational processes of consumers to orient the marketing mix (e.g., target-appropriate audiences and markets, increasing the effectiveness of ads and products) [110]. According to previous studies, NM research has used the approach/withdrawal attitude to evaluate TV ads [111]. Therefore, approach/withdrawal motivational attitudes are highly significant for marketing and advertising research.

##### Reward Processing

According to the literature, it is highly significant for researchers and practitioners to study and know the neural responses that are responsible for reward processing, such as money, food, and social activities [112,113,114,115]. This is because the positive rewards such as gaining money, foods, or other types of rewards, enhance the accuracy and cognitive task [116,117,118] through modifying the early attentional process. Anderson, et al. [101] demonstrated that visual features (e.g., product design) that are correlated to reward, will make the consumer prioritize, therefore attracting the consumer’s attention automatically. For example, the design preference of a product or brand can increase the activity in the regions which are responsible for reward processing, thereby, causing more activation in regions of motivations that might impact consumers’ purchase decisions [29]. Many studies concentrated on the individual’s response toward a monetary reward by studying the approach/avoidance attitude [112,113]. For example, Bechara, et al. [119] carried out an experiment named the “Iowa Gambling Task” by using GSR to investigate the influence of reward on decision-making. They divided participants into two groups, the healthy group and the group with lesions in the vmPFC. The findings revealed that healthy participants sweated more, which led them to infer that participants had a negative emotional experience toward picking up cards from a monetary losing deck; meanwhile, the lesion group picked up cards regardless of whether they were cards with monetary wins or losses. Consequently, reward highly influenced decision-making [112,120,121].

Many researchers have confirmed that the importance of the striatum activity in reward processing, wherein the components of the striatum such as the caudate nucleus, nucleus accumbens (NAcc), and putamen play a central role in expectation and evaluation of reward [115,122,123]. For example, Galvan [124] and Geier, et al. [125] carried out an experiment to investigate the relationship between reward processing and the striatum. Their findings revealed that the ventral striatum (VS) has a key role in the prediction of reward. Jung, et al. [126] found that the rewards, memory, semantics, and attention regions in the brain were lit up when viewing a combination of a celebrity face and a car, compared with viewing a combination of a non-celebrity face and a car. In addition, car favourableness correlated positively with activation in the left anterior-insula, left OFC, and left higher-order visual cortex in the OL. Padmanabhan, et al. [127] investigated the influence of the reward system on attention processes. Their findings showed that incentives facilitate cognitive control. Previous neuroimaging studies demonstrated that rewards activate the ventral medial prefrontal cortex (vmPFC) and ventral striatum [128,129,130]. The ventral striatum has been discussed before as a part of the reward system [131]. Therefore, findings suggest that neurodevelopmental changes in the striatum systems may contribute to changes in how reward influences attentional processes [56].

##### Attention

Attention is defined as the way “people tend to seek, accept and consume the messages that meet their interests, beliefs, values, expectations and ideas, and overlook the messages that are incompatible with this system” [132]. It has also been defined as selective perception [133]. Selective perception is associated with filtering out information and concentrating on significant information (e.g., different aspects of stimulus or different stimuli) [134]. For instance, consumers are exposed to nearly 10 million bits of visual information (e.g., ads, images, sound, video, and colour) per second through their senses (e.g., eyes, ears, skin) daily. Most input information goes by unnoticed, with consumers able to process almost 40 bits of input information per second [29,135]. This lead us to infer that attention has a strong influence on consumer behaviour in how consumers represent, perceive and process information and thus select and prioritize information. Attentional and emotional processes are relatively intersected/connected, and emotion is considered a reliable and effective source for attracting consumers’ attention [136,137]. For example, the activation in the amygdala (AMY) and cingulate cortex (CC) in the brain is related to emotional stimuli.

**Figure 4:**
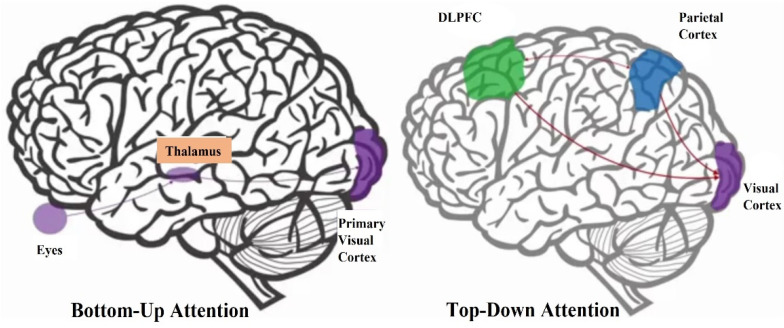
The bottom-up and top-down attention processes [59].

Attention is a significant brain process, which has a central role in measuring the effectiveness of advertising campaigns; thereby, it is an indicator of consumer’s behaviour and the effectiveness of advertising [138]. According to the literature, the majority of researchers have agreed on two systems to measure attention toward advertising: (i) bottom-up, and (ii) top-down, systems [28,139,140]. Bottom-up (visual saliency/exogenous/ involuntary) attention is the type of attentional system which is initiated by external stimuli such as colour, voice, promotion, faces, text, novelty, brightness, and so forth, which lead to a process in which information in external stimuli is received automatically. Top-down (goal-driven/endogenous/voluntary) attention is the other type of attentional system, which is initiated by internal and external goals and expectation; thereby, it is necessary to focus all your mental power toward the goal that you are looking to achieve, thereby filtering goals to achieve your goals (Figure 4) [2,140,141].

For this reason, the underlying brain reactions of attention and visual processing are highly interesting for advertising. Moreover, the anterior cingulate cortex (ACC) is highly related to the function of top-down and bottom-up attentional systems [142,143]. For example, Smith and Gevins [144] found that the occipital lobe (OL) is associated with attentional processes toward TV advertisements. The EEG investigation of Kong, et al. [145] found that variation in activity in the cerebral hemisphere related to the cognitive task can help to determine the success or lack of success of the advertisement. A recent fMRI investigation by Casado-Aranda, et al. [146] found that the correspondence between advertising and gender voice (male, female) induces attention regions in the brain. Ananos [147] carried out experiment using ET to investigate the attention level and processing of information in advertising (content recognition) among elderly and young people groups. Their findings revealed that the attention level among both groups was the same, but recognition by the young-people group was higher than that of the elderly-people group for untraditional advertising. Guixeres, et al. [51] conducted an experiment to investigate the effectiveness of an ad (e.g., a recall ad) and the number of views on YouTube channels, using neural networks and neuroscience-based metrics (e.g., brain response, ECG, and ET). Their findings suggest an important relationship between neuroscience metrics and self-report of ad effectiveness (e.g., recall ad) and the number of views on YouTube. Cuesta-Cambra, et al. [148] investigated how information is processed and learned and how visual attention takes place. Their findings indicated that the visual activity of men has different patterns from women, and does not influence subsequent recall, wherein recall relies on the emotional value of ads and simplicity, while complex ads need more visual fixation and are therefore hard to remember. In addition, the importance of the playful component of memory and low-involvement processing were confirmed by EEG. Treleaven-Hassard, et al. [149] examined the engagement of the consumer with interactive TV ads with a particular brand’s logo compared with non-interactive TV ads. The findings revealed that brands linked with interactive ads attract more automatic attention. Boscolo, et al. [81] conducted an experiment using ET and questionnaires to investigate differences in the visual attention between genders (male and female), toward print ads. Their findings revealed that there is difference in visual attention in the case of male, while no differences were noticed in the case of females.

##### Perception

**Figure 5:**
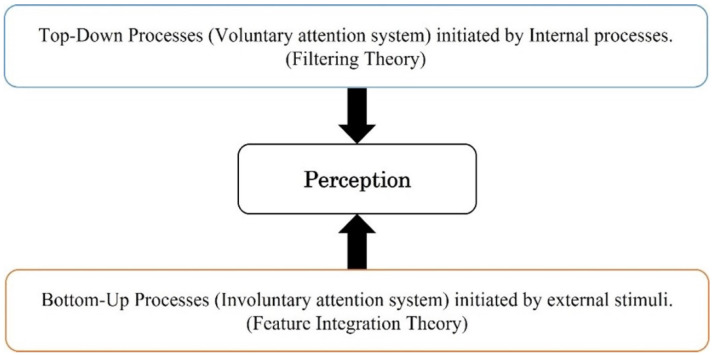
Two attentional systems impact the consumers’ perceptions.

According to Simson [150], studies into the perception of value and how it is formed reflect what is known in marketing theory, wherein the marketing-mix elements can be changed to influence the perceived value of a product. However, studies on how attention systems impact consumers’ perception and actions have been limited to consumer report and behavioural studies, which depend on a rational report; this is not enough to explain attention processes, wherein there are two attentional systems influencing consumers’ perceptions (e.g., top-down and bottom-up attention system) (Figure 5) [59]. Consumer perception is the first step in engagement with marketing stimuli or any other stimuli in the environment [151]. Hogg, et al. [152] defined perception as “the process by which marketing stimuli are selected, organised, and interpreted”. Therefore, individuals add meaning and interpret it in a certain way, which leads to the perceptions of the individual’s findings for each one. As stated by Belch and Belch [153] perception processing is extremely reliant on internal processes such as prior knowledge (experiences), current goals, beliefs, expectations, needs, and moods, and also external stimuli such as colour, orientation, intensity, and movement [59]. Although this explains the process of how consumer perceptions are formed, the exact the part concerning the explanation of sensations and the internal and unique assignation of meaning to sensations is what lies concealed, and remains unexplained in detail in the current consumer-behaviour literature. However, it is widely believed that this process is driven by the unconscious.

Cartocci, et al. [154] and Modica, et al. [155] conducted experiments to estimate the accuracy measurement of the cerebral and emotional perception of social advertising campaigns (i.e., antismoking) using several methods such as EEG, GSR, and ECG. The findings showed that the antismoking campaign which was characterized by a symbolic communication style gained the highest approach-values, as evaluated by the approach/withdrawal index. Meanwhile, an image based on “fear-arousing appeal” and with a narrative style reported the highest and lowest effort-values index, respectively. The fMRI investigation of Falk, et al. [156] predicted the out-of-sample (population) effectiveness of quit-smoking ads. The findings revealed that activity in the prior mPFC was largely predictive of the success of various advertising campaigns in the real world. Plassmann, et al. [157] carried out an experiment to study the perception of pleasantness in the taste of wines, using the fMRI tool. Their findings showed a stronger activation in the medial OFC (mOFC) regions in the brain when subjects believed they are drinking expensive wine, showing that the mOFC is responsible for experiencing pleasantness. This led to infer that the pleasantness report was correlated with perceived value and price of product more than taste itself. Neuroscientists have found that the OFC and ventromedial prefrontal cortex (vmPFC) are involved in decision-making, through the perceived value of products [158]. Nuñez-Gomez, et al. [159] carried out an experiment using EEG to examine how advertising material is perceived by two groups (e.g., healthy group and group with Asperger syndrome). The findings revealed that there are large difference between these groups in their perception of emotion and their attention variables. Gong, et al. [160] carried out an experiment to identify the influence of sales promotion (e.g., gift-giving, discount) on the perception of consumers and purchase decisions by using EEG/ERP. The findings revealed that discount promotions have more impact on purchase decisions than gift-giving sales promotions.

##### Memory

**Figure 6:**
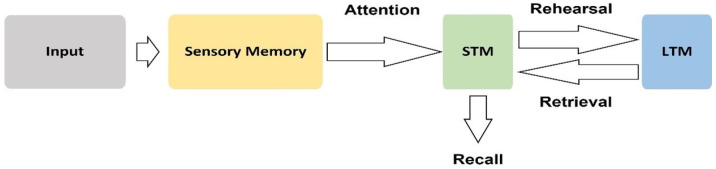
The information phases in the memory’s stages [164].

Memory is defined as an ongoing learning-process, which has input and output functions in the brain [161,162]. The input function encodes information, while the output function retrieves information, and this is very important for advertising research [137,163]. For example, recall and recognition advertising-information is a retrieving function [28]. Myers and DeWall [162] and Atkinson and Shiffrin [163] presented the multistore model of memory, which includes three steps, as follows: (i) a sensory memory, (ii) short-term memory (STM), and (iii) long-term memory (LTM) (Figure 6) [164]. Brain processes related to memory have revealed certain valuable outcomes, as to the factors which influence the consumers’ behaviour ,such as recall- and recognition-advertising [165]. Input and output functions in the memory are highly important for marketers and advertisers, due to each function having an awareness and unawareness aspect in the brain [137,166]. Memory and emotion are highly connected to each other. For example, previous studies confirmed that the emotional events are usually remembered more than neutral events, and especially if emotions correspond to events at that moment [167].

The memory process has been widely studied, and it has concluded that the hippocampus (HC), located in the temporal lobe (TL), plays a major role in generating and processing memories [165]. Additionally, activation of the HC has a strong relationship with LTM and STM, which highly impacts consumers’ purchase decisions [168,169]. In addition, the AMY is located next and close to the HC, which is significant for the memory system [165]. For example, the EEG investigation by Rossiter, et al. [170] found that the left hemisphere is correlated with encoding in the LTM. Similarly, the EEG investigations by Astolfi, et al. [171] used EEG to determine the brain regions that were triggered by the successful memory-encoding of TV ads. They found stronger activity in the cortical regions. Morey [172] investigated the impact of advertising message on recognition memory. The findings revealed stronger activity in the gamma band, which directly affected memory. The fMRI investigation by Bakalash and Riemer [173] and Seelig, et al. [174] measured the brain regions of memory ads. The findings revealed that stronger activity in the amygdala (AMY) and frontotemporal regions is associated with memorable and unmemorable ads. Similarly, [175] carried out experiments to investigate the content of ads and the activity of frontal regions and memory. The findings showed that the content of ads increased the activity in the frontal regions and the input function (encoding) of memory.

The study of these mental processes such as emotion and feelings, attention, memory, reward processing, motivation, and perception are highly important considerations for advertising research.

## 4. Discussion

A total of 76 articles were extracted and analysed, wherein the content analysis of the relevant articles revealed that the annual and the accumulative number of publications has been growing since 2009, reaching its peak in 2020 with twelve empirical articles that used neuroimaging, physiological, and self-report techniques to study the consumers’ brain processes such as, but not limited to, emotions toward the stimuli of marketing such as advertising. We followed the PRISMA protocol to select the relevant empirical articles for this study as brain processes such as emotions, feelings, motivation, reward, attention, and memory need to be considered in advertising research. The findings of the study revealed that the neuroimaging tool that is used most in studying the brain processes of consumers is the EEG, with 38 empirical articles, followed by the fMRI with 20 articles; it was also noticed that only four articles used the fNIRS tool in neuromarketing research. In addition, for physiological tools, it was observed that five techniques were used in neuromarketing studies to investigate consumer responses toward the stimuli of marketing such as advertising. The ET was used in 14 articles alongside neuroimaging tools such as EEG, wherein ET is the most used tool, followed by GSR with 12 articles; it was also used alongside other physiological tools such as ECG and EMG. Finally, self-report (i.e., surveys, interviews, focus groups, and observation) was used in seven articles.

This study found that the brain processes to be considered most in advertising research are emotions, feelings, attention, memory, perception, approach/withdrawal motivation, and reward processing. The findings demonstrated that the strongest activity in the inferior-frontal- and middle-temporal-gyri are associated with pleasure and displeasure, while the activity in the right superior-temporal and the right middle-frontal-gyrus correlated with high or low arousal [90]. As well as this, we found the OL connected with the attention system [144], and the HC, located in the temporal lobe (TL), plays a major role in generating and processing memories [165]. In addition, the VS, which is located in the basal ganglia plays a central role in the reward system; for example, the key functions of VS (i.e., control movement and planning) have a vital role in the reward system, while the components of VS such as the putamen, caudate nucleus, and nucleus accumbens (NAcc) have a central role in the assessment of consumer expectations, compared to the actual reward received [123]. In addition, the ventral tegmental region is considered a part of the reward system, which passes the neurotransmitter dopamine to other brain areas, thereby affecting goal-seeking behaviour [123]. For motivation, it was found that the anterior cerebral hemispheres play a central role in withdrawal and approach motivation; for example, the increase in activation in the right PFC is linked to withdrawal behaviour, while the increase in activation in the left PFC is associated with approach behaviour [105,176]. Finally, in accordance with the literature, it was found that the OFC and vmPFC regions play vital roles in perception (i.e., perceived value) [158].

## 5. Conclusions and Implications

***Implication of the research findings for theory and practice:*** Theoretically, the current findings can be divided into three areas, as follows: firstly, neuroscientific techniques and methods enable the capture/measurement of the activity signals of the brain and body relating to consumers’ responses (e.g., emotions and feelings, attention, memory, perception, reward processing, and motivation) toward advertising campaigns. For example, neuroimaging tools (e.g., fMRI, EEG/ERP, fNIRS) enable the recording of the neural signals of the mental responses (e.g., pleasure/displeasure, low/high arousal, advertising recall and recognition) toward advertising, which can be beneficial for advertisers and marketers in creating more effective advertising campaigns to attract, captivate, and impact consumers. Meanwhile, physiological tools (e.g., ET, GSR, EMG, and ECG) enable researchers to gauge the physiological reactions of the consumer, such as pupil dilation, fixation, eye movements, saccade, heart rate, blood pressure, sweating level, and reaction time toward advertising. Secondly, neuroimaging and physiological tools will help advertisers and scholars to identify the weak elements in advertising which lead to withdrawal behaviour and to address these, besides identifying the strengths which lead to approach behaviour, and to enhance them. Thirdly, many articles have concentrated on detecting the neural and physiological responses of emotions, feelings, attention, memory, reward processing, motivation and perception toward advertising such as the presenter’s features (i.e., celebrity), because these processes play a key role in the decision-making of consumers (i.e., purchasing decisions). Additionally, some advertising research concentrated on gender voice (i.e., male, female), ads appeal, faces of celebrity, social campaigns (i.e., using seat belts in the car), and public health (i.e., anti-smoking campaigns). These areas can provide a reasonable explanation of the neural and physiological correlates of emotions and feelings (e.g., pleasure/displeasure, high/low arousal), attention (e.g., top-down, bottom-up), memory (e.g., encoding, retrieving), motivation (e.g., approach/withdrawal), reward processing, and perception (e.g., perceived value of ads) to be considered in advertising research. Thus, an application of this research perhaps offers reasonable explanations of how advertising works in consumers’ minds, and the relationship between the neural correlates of consumers’ brain and physiological responses toward advertising, thereby better understanding consumers’ behaviour, which leads to the creation of more attractive advertising for political, social and business sectors.

***General Conclusion***: Neuromarketing is a promising field, not only to study and solve the commercial issues such as the weaknesses of advertising campaigns and to reduce the wastage of advertising budgets, but also to create more effective advertising campaigns in social, political, and public-health sectors, in order to increase human awareness. In today’s hyper-competitive environments among advertising agencies, each agency seeks to find the most beneficial methods to beat competitors and be the first priorities in the consumer’s mind. Thus, advertisers have adopted neuroscientific methods in their research to study, analyse, and predict the neural and physiological responses of consumers toward the stimuli of marketing (i.e., advertising), thereby identifying the most important mental and physiological responses to be considered in advertising research to raise advertising effectiveness. Most studies in advertising research have determined the main mental processes of interest for advertising research, such as emotions and feelings, attention, memory, reward processing, motivation, and perception.

The findings of the study suggest that neuroscientific methods and techniques are significant to gauge the brain and physiological reactions of consumers toward the stimuli of marketing, such as advertising research. For example, neuroimaging tools are able to gauge the neural-activity signals of the consumer’s brain. At the same time, physiological tools can gauge physiological reactions such as eye movements, sweating level, and fixation.

## 6. Limitations and Future Directions

This paper tried to minimize the limitations in methodology; however, some limitations occurred and provided several directions for further research. This research concentrated on the English articles that were published in open-access journals from 2009 to 2020 and were listed in the WOS database. Therefore, this paper overlooked non-English articles, non-open-access articles, and other documents such as books, review papers, conference proceedings, and so forth. Thus, this paper is not free of bias. For future directions, we hope to overcome the obstacles in the future, which include the cost of research and techniques, lack of labs and facilities, use of time (e.g., data interpretation, recruiting participants, and so forth), and increased investment and funding in neuromarketing research and technique [177]. We encourage researchers and marketers from emerging countries to enter this embryonic field and leave their footprint by publishing articles for future works. In addition, we suggest that researchers and scholars identify the influence of advertising on consumers persuasion, engagement, and excitement, as well as the contributions of neuromarketing research to various domains (e.g., social sciences, public health, politics, and stock exchanges). We believe that this review study provides a profound overview of the global academic-trends in neuromarketing research, using the neuroimaging and physiological studies in advertising to study the brain processes of consumers. Thus, it provides valuable and reliable insights into the appropriate brain processes to be considered in future research.

## Figures and Tables

**Table 1:** Classifications of neuroimaging and physiological tools used in advertising research.

Classifications	Tool	No. of Studies	Percentage (%)
Neuroimaging tools	EEG	38	50%
	fMRI	20	26.3%
	fNIRS	4	5.3%
Physiological tools	ET	14	18.4%
	GSR	12	15.8%
	ECG/HR	9	11.8%
	IAT	4	5.3%
	EMG	3	4%
Self-report	Surveys, interviews, observation	7	9.2%

**Table 2:** Neuromarketing tools to measure emotions and feelings.

Emotional Processes	Dimensions	Classification	Tools to Measure Emotions and Feelings
Emotions	Valence, Arousal	Neuroimaging tools	fMRI, PET, EEG, SST, EMG
		Physiological tools	SST, GSR/SC
Feelings		Self-reports	Surveys, interviews, focus groups, and observation

## Data Availability

Not applicable.
